# Clinical Efficacy of Nutritional Intervention Combined with Muscle Exercise on Sarcopenia Patients with Femoral Fracture: A Pilot Randomized Controlled Trial

**DOI:** 10.1155/2023/3222686

**Published:** 2023-02-10

**Authors:** Na Nie, Zhihua Yin, Jiao Zheng, Xiaoqian Lin, Yu Chen, Xuping Xie, Xinyue Ding, Pei Li, Jian Yang

**Affiliations:** ^1^Department of Orthopedics, The Third Affiliated Hospital of Chongqing Medical University, Chongqing 400010, China; ^2^Department of Orthopedics, The First Affiliated Hospital of Chongqing Medical University, Chongqing 400010, China; ^3^Department of Nutriology, The Third Affiliated Hospital of Chongqing Medical University, Chongqing 400010, China

## Abstract

**Objective:**

To study the clinical efficacy of nutritional intervention combined with muscle exercise on sarcopenia patients with femoral fracture.

**Methods:**

From January 2019 to January 2021, a total of 100 sarcopenia patients with femoral fracture were included in this study and were divided into a control group (routine postoperative care) and a research group (nutritional intervention and muscle exercise), 50 cases in each group. Primary clinical outcomes included sarcopenia-related indicators and functional independence assessed by activities of daily living scale (ADL). Secondary clinical outcomes included time of fracture healing and hospital stay, pain score as assessed by visual analogue scale (VAS), and nursing satisfaction.

**Results:**

Before the intervention, there was no significant difference in the indicators of sarcopenia and the indicators of functional independence assessed by ADL between the two groups (*P* > 0.05). After 3 months of intervention, the BMI, grip strength, calf circumference, pace, and body muscle rate of the patients in the research group were significantly higher than those in the control group (*P* < 0.05), while body fat rates were significantly lower than those in the control group (*P* < 0.05), and the capability of eating, walking, bathing, and doing housework in research group were all significantly higher than those in control group (*P* < 0.05). In addition, the time of fracture healing and hospital stay in research group were all significantly lower than those in control group (*P* < 0.05), and the VAS scores of the control group at each time point after intervention were significantly higher than those of the research group (*P* < 0.05). The nursing satisfaction of the patients in the research group was significantly higher than that in the control group (94.00% vs. 76.00%, *P* < 0.05).

**Conclusion:**

Nutritional intervention combined with muscle exercise can help improve sarcopenia symptoms and promote fracture recovery in patients with sarcopenic femoral fractures.

## 1. Introduction

Sarcopenia is a syndrome of persistent loss of skeletal muscle mass, strength, and function and characterized by a marked age-dependent onset. Previous studies have found that skeletal muscle begins to age from about 40 years, with an average annual decrease in quantity and quality of about 8%, and muscle loss doubles after the age of 70 [1, 2]. Epidemiological statistics on sarcopenia show that the prevalence of sarcopenia is less than 20% in people under 70 years old, about 30% in people 70-80 years old and more than 80 years old in people over 80 years old [3, 4]. Skeletal muscle is the most important motor organ of human, the largest protein storage and an important glucose metabolism organ, so the reduction of skeletal muscle will cause malnutrition and immune system damage, thereby increasing the risk of other diseases in the elderly [5]. Sarcopenia is often associated with osteoporosis, collectively referred to as the “mobility disorder syndrome,” which predisposes the elderly to falls and fractures and most commonly hip and femur fractures [6, 7].

Importantly, fracture patients need to stay in bed for a long time, and long-term bed rest is more prone to muscle strength loss caused by muscle reduction, and the probability of falling when getting out of bed is very likely to increase, thereby increasing the risk of femoral fracture again [8, 9]. Therefore, reducing the occurrence of sarcopenia, preventing falls in patients with fractures, and promoting the recovery of limb function are an important part of orthopaedic nursing work. Nutritional support, muscle building, and drug therapy are considered effective measures to prevent and treat sarcopenia [10, 11]. However, a previous study has found that nutritional support alone cannot effectively improve muscle mass and functional status in patients with sarcopenia [12], while nutritional intervention combined with physical exercise has a significant effect on improving muscle mass and function in patients with sarcopenia [13, 14].

Femoral fracture is one of the common diseases in clinical surgery, characterized by severe pain, swelling, and distortion, which have a serious impact on the patient's ability to live. Sarcopenia and osteoporosis are two major risk factors for fractures in patients, especially elderly patients [6, 7]. In this study, we design to investigate the clinical efficacy of nutritional intervention combined with muscle exercise on sarcopenia patients with femoral fracture.

## 2. Material and Methods

### 2.1. Patients and Ethics Statement

A total of 100 patients with sarcopenic femoral fractures were included in this study from January 2019 to January 2021 (age from 57 to 74 years old, 53 males and 47 females). The following are the inclusion criteria: (1) femoral fracture and received artificial joint replacement; (2) combined with sarcopenia; (3) aged 40-75 years; (4) mentally stable, able to communicate normally and to move independently, and voluntarily cooperate with this study. The following are the exclusion criteria: (1) complicated with severe cardiopulmonary insufficiency; (2) severe neuropsychiatric disorders; (3) inability to stand up independently; (4) impaired cognitive function; (5) severe renal insufficiency requires protein intake to be restricted; (6) patients with malignant disease or infectious disease; and (7) lost in the middle of the study, suffering from other diseases or death. All patients were informed about the study and signed the informed consent to join the study voluntarily. In addition, this study was a prospective study; the present study was approved and supervised by The Third Affiliated Hospital of Chongqing Medical University Hospital Ethics Committee.

### 2.2. Intervention Protocol

Patients in the control group were received routine care such as primary care and symptomatic care. Patients in the research group received nutritional interventions and muscle exercises in addition to routine care. Nutritional intervention and muscle exercise duration is from 10 days postoperatively to 3 months plus 10 days postoperatively. The following are the nutritional interventions: daily supplementation of 400 kcal of whole nutritional formula powder and 0.6-1.0 g/kg of whey protein powder. The following are the muscle exercises: do 30 minutes of aerobic and resistance exercise each time, 5 times a week, including brisk walking and slow walking. Muscle exercises are guided by medical staff, mainly targeting the leg muscles, with video instruction which is given once a week.

### 2.3. Data Collection and Follow-Up

Baseline data were collected for all patients recruited in this study, including gender, age, education, comorbidities (hypertension, diabetes, and cardiopulmonary disease), drinking, and smoking. All patients received a 4-month follow-up after surgery, including telephone follow-up every 2 weeks and unscheduled hospital admissions.

### 2.4. Primary Clinical Outcome

Primary clinical outcomes included sarcopenia-related indicators and functional independence assessed by activities of daily living scale (ADL) [15]. In detail, before the intervention and 3 months after the intervention, researchers measured sarcopenia-related indicators, including body mass index (BMI), grip strength, pace, body muscle percentage, and body fat percentage in all patients: (1) BMI = weight (kg)/height^2^(m^2^); (2) in grip strength, the subject stands and relaxes, with both hands hanging down naturally; hold the grip dynamometer with one hand, and firmly clench the grip dynamometer once, and record the grip strength; (3) in pace, record the time spent walking 6 m, and calculate the pace; and (4) flex the knee joint 90, and measure the thickest part of the calf. Moreover, we used activities of daily living scale to evaluate functional independence of patients before the intervention and 3 months after the intervention. ADL scale evaluates the capability of eating, walking, bathing, and doing housework in patients, and the higher the score, the stronger the functional independence.

### 2.5. Secondary Clinical Outcome

Secondary clinical outcomes included the time of fracture healing and hospital stay, pain as assessed by visual analogue scale (VAS) at different times, and patient satisfaction with care. In detail, we used VAS to assess pain before intervention, 1 week, 2 weeks, 1 month, and 3 months after intervention. The VAS scoring standard is divided into 10 equal parts using a ruler, 0 is no pain, 1-3 is mild pain, 4-6 is moderate pain, and 7-10 is severe pain.

### 2.6. Statistical Analysis

Data in the present study were analyzed by SPSS 20.0 software (SPSS Inc., Chicago, USA). Qualitative data are presented as counts (%), and *P* values are calculated using a chi-square or Fisher's exact test as appropriate. The Kolmogorov-Smirnov test was used to check whether quantitative data conformed to a normal distribution, data that conformed to a normal distribution were presented as (mean ± standard deviation), and unpaired Student's *t*-test was used to compare differences and calculate *P* values. *P* < 0.05 indicated significant difference.

## 3. Results

### 3.1. Baseline Data

167 patients were enrolled in the beginning, and 67 were excluded, and no patient lose to follow-up. 100 eligible patients were randomly divided into two groups, control group and research group. There were no significant differences between the two groups in baseline data (*P* > 0.05), including gender, age, education, comorbidities (hypertension, diabetes, and cardiopulmonary disease), drinking, and smoking (Table 1).

### 3.2. Sarcopenia Evaluation Related Indicators

There was no significant difference in the indicators of sarcopenia between the two groups before the intervention (*P* > 0.05), including BMI, grip strength, calf circumference, pace, body muscle percentage, and fat percentage (Table 2). After the intervention, the BMI, grip strength, calf circumference, walking speed, and body muscle rate of the patients in the research group were significantly higher than those in the control group (*P* < 0.05), while body fat rate were significantly lower than those in the control group (*P* < 0.05) (Table 2).

### 3.3. Functional Independence

There was no significant difference in the functional independence between the two groups before the intervention (*P* > 0.05), including the capability of eating, walking, bathing, and doing housework (Table 3). After the intervention, the capability of eating, walking, bathing, and doing housework in patients of the research group were all significantly higher than those of the control group (*P* < 0.05) (Table 3).

### 3.4. Postoperative Recovery

The time of fracture healing and hospital stay in patients of research group were all significantly lower than those in patients of control group (*P* < 0.05) (Table 4). However, there was no significant difference between the two groups on the VAS score before intervention, while the VAS scores of patients in research group at 1 week, 2 weeks, 1 month, and 3 months after intervention were all significantly lower than those in patients of control group (*P* < 0.05) (Table 4).

### 3.5. Nursing Satisfaction

The number of patients in the control group was 16, 22, and 12 who were very satisfied, satisfied, and dissatisfied with their care, compared with 24, 23, and 3 in the research group, respectively. The patients in the study group had a satisfaction rate of 94.00%, which was significantly higher than that of the patients in the control group (76.00%) (*P* < 0.05) (Table 5).

## 4. Discussion

Sarcopenia, an age-dependent loss of muscle mass and function, is a common disorder in older adults and is associated with adverse health outcomes. Sarcopenia patients suffer from loss of muscle strength and mobility impairments, which reduce their quality of life, with higher risk for falls, fractures, metabolic disease, and mortality [5]. In China, a survey of 6172 individuals aged 60-94 found that the prevalence of suspected sarcopenia, sarcopenia, and severe sarcopenia was 38.5%, 18.6%, and 8.0%, respectively [16]. In addition, previous studies also showed that patients with sarcopenic fractures had a significantly increased risk of incomplete postoperative recovery and lower Barthel index at discharge [17], longer hospital stays [18], and higher mortality than nonsarcopenic patients [19, 20]. Importantly, the prevalence of osteoporosis increases with the severity of sarcopenia, which together increase fracture risk in older adults and those recovering from fractures. Therefore, it is necessary to increase the awareness of sarcopenia in fracture patients and pay attention to the occurrence or progression of sarcopenia during the rehabilitation of fracture patients [6, 7].

In this study, we found that compared with patients in the control group, patients in the research group receiving nutritional intervention combined with muscle exercise had higher BMI, grip strength, calf circumference, faster walking speed, and body muscle rate after the intervention, which suggests that nutritional intervention combined with muscle exercise can effectively improve symptoms in patients with sarcopenia. Undernutrition is an important cause of sarcopenia in middle-aged and elderly people, and it is also the main direction for the prevention and treatment of sarcopenia [21, 22]. Malnutrition associated with sarcopenia begins with insufficient protein intake. In Europe, up to 10% of older adults living in the community and 35% of older adults in institutional care do not meet the minimum protein intake (0.7 g/kg bw/day) required to maintain muscle integrity in adults of all ages [23, 24]. In the United States, 32% to 41% of women and 22% to 38% of men in the age group 50 years and older consume less than the recommended protein intake of 0.8 g/kg bw/day [25]. Herein, our nutritional intervention protocol is daily supplementation of 400 kcal of whole nutritional formula milk powder and 0.6-1.0 g/kg of whey protein powder, which is borrowed from many previous studies [26, 27] and confirmed by the results of this study for sarcopenic femoral fractures patients are effective.

Furthermore, muscle exercise is also very important for improving symptoms in patients with sarcopenia. On the one hand, regular aerobic training can improve the activity of muscle mitochondrial enzymes, increase capillary density, promote nerve repair and motor unit mobility, thereby increasing skeletal muscle strength and exercise capacity [28, 29]. On the other hand, regular resistance exercise can maintain and improve the muscle strength quality of middle-aged and elderly people [30, 31]. Therefore, muscle exercises including aerobic and resistance exercises were included in the intervention protocol for patients in the research group.

In this study, we also found that the postoperative recovery of the patients in the research group was better than that of the patients in the control group, including higher functional independence scores, shorter fracture healing times and hospital stay time, and lower postoperative VAS pain scores, which indicates that our intervention not only improves sarcopenia symptoms but also accelerates recovery in patients with femoral fractures. Adequate nutrition and protein supplementation can not only slow down muscle atrophy but also help increase muscle strength and help patients with femoral fractures get out of bed early to exercise [32, 33]. Early activity and early walking can increase the patient's appetite and increase the intake of nutrients such as protein, which has a significant effect on reducing the occurrence of complications and promoting functional recovery [34, 35]. Therefore, our intervention protocol helps to create a virtuous circle between improvement in sarcopenia-related symptoms and accelerated recovery from femoral fractures.

All in all, our results in this study indicated that nutritional intervention combined with muscle exercise could help improve sarcopenia symptoms and promote fracture recovery in patients with sarcopenic femoral fractures. However, as a single center study, there are problems such as a single research population and a small sample size. In the future, we need to expand the sample size to verify the accuracy of the results.

## Figures and Tables

**Table 1:** Baseline data in two groups.

Groups	Control group (*n* = 50)	Research group (*n* = 50)	*t*/*χ*^2^	*P*
Male/female (*n*)	28/22	25/25	0.361	0.548
Age (years, mean ± SD)	67.28 ± 5.13	66.76 ± 5.15	0.506	0.614
Hypertension (*n* (%))	32 (64.00)	35 (70.00)	0.407	0.523
Diabetes (*n* (%))	13 (26.00)	18 (36.00)	1.169	0.280
Cardiopulmonary disease (*n* (%))	16 (32.00)	17 (34.00)	0.045	0.832
High school and below (*n* (%))	40 (80.00)	42 (84.00)	0.271	0.603
Drinking (*n* (%))	12 (24.00)	13 (26.00)	0.053	0.817
Smoking (*n* (%))	15 (30.00)	16 (32.00)	0.047	0.829

**Table 2:** Comparison of sarcopenia-related indicators in two groups of sarcopenic femoral fracture patients before and after treatment (mean ± SD).

Groups	Time point of intervention	Control group (*n* = 50)	Research group (*n* = 50)	*t*	*P*
BMI (kg/m^2^)	Before	19.55 ± 1.64	19.83 ± 1.50	0.901	0.370
	After	20.56 ± 1.74	21.96 ± 1.66	4.019	<0.001

Grip (kg)	Before	19.06 ± 1.15	18.56 ± 2.73	1.192	0.188
	After	19.59 ± 1.22	20.92 ± 3.01	2.903	0.005

Calf circumference (cm)	Before	31.31 ± 2.46	31.41 ± 2.56	0.207	0.836
	After	32.01 ± 2.58	35.76 ± 2.81	6.955	<0.001

Walking speed (m/s)	Before	0.74 ± 0.12	0.75 ± 0.13	0.366	0.715
	After	0.75 ± 0.12	0.94 ± 0.16	6.595	<0.001

Muscle rate (%)	Before	39.76 ± 1.76	39.65 ± 1.30	0.362	0.718
	After	39.13 ± 1.68	41.68 ± 1.36	8.339	<0.001

Fat percentage (%)	Before	23.50 ± 0.88	23.66 ± 1.06	0.842	0.402
	After	25.92 ± 1.11	23.46 ± 1.04	11.375	<0.001

**Table 3:** Comparison of functional independence between the two groups of patients (mean ± SD).

Groups	Time point of intervention	Control group (*n* = 50)	Research group (*n* = 50)	*t*	*P*
Eating	Before	32.28 ± 0.99	32.28 ± 1.18	0.745	0.390
	After	45.08 ± 1.41	59.70 ± 4.19	52.070	<0.001

Walking	Before	32.32 ± 1.00	32.10 ± 2.13	0.661	0.510
	After	46.68 ± 1.41	59.70 ± 4.19	20.828	<0.001

Bathing	Before	35.34 ± 1.10	35.32 ± 1.30	0.083	0.934
	After	49.32 ± 1.54	60.34 ± 2.04	30.487	<0.001

Doing housework	Before	32.22 ± 1.15	31.94 ± 2.66	0.683	0.496
	After	41.98 ± 1.49	49.80 ± 3.87	13.347	<0.001

**Table 4:** Comparison of postoperative recovery between the two groups of patients (mean ± SD).

Groups	Control group (*n* = 50)	Research group (*n* = 50)	*t*	*P*
Fracture healing time (week)	12.50 ± 1.57	10.66 ± 1.44	6.116	<0.001
Hospital stay time (day)	15.00 ± 1.46	12.38 ± 1.50	8.870	<0.001
VAS score in different time				
Before intervention	5.80 ± 1.52	5.72 ± 1.05	0.030	0.646
1 week after intervention	5.50 ± 1.41	4.80 ± 0.99	4.096	<0.001
2 weeks after intervention	4.90 ± 1.15	4.40 ± 0.81	2.519	0.013
1 month after intervention	2.72 ± 0.86	1.90 ± 1.15	4.407	<0.001
3 months after intervention	1.70 ± 0.46	0.40 ± 0.49	13.565	<0.001

**Table 5:** Comparison of nursing satisfaction of two groups of patients (*n* (%)).

Groups	*n*	Very satisfied	Satisfied	Dissatisfied	Satisfied rate (%)
Control group	50	16 (32.00)	22 (44.00)	12 (24.00)	76.00
Research group	50	24 (48.00)	23 (46.00)	3 (6.00)	94.00
*χ* ^2^					6.352
*P*					0.012

## Data Availability

All data of this study are available from the corresponding author upon request.
